# Risk Factors for Venous Thromboembolism After Spine Surgery

**DOI:** 10.1097/MD.0000000000000466

**Published:** 2015-02-06

**Authors:** Hiroyuki Tominaga, Takao Setoguchi, Fumito Tanabe, Ichiro Kawamura, Yasuhiro Tsuneyoshi, Naoya Kawabata, Satoshi Nagano, Masahiko Abematsu, Takuya Yamamoto, Kazunori Yone, Setsuro Komiya

**Affiliations:** From the Department of Orthopaedic Surgery, Graduate School of Medical and Dental Sciences, Kagoshima University, 8-35-1 Sakuragaoka, Kagoshima 890-8520, Japan (HT, FT IK, SN, MA, TY, SK); The Near-Future Locomotor Organ Medicine Creation Course (Kusunoki Kai), Graduate School of Medical and Dental Sciences, Kagoshima University, 8-35-1 Sakuragaoka, Kagoshima 890-8520, Japan (HT, TS); Department of Orthopaedic Surgery, Izumi Regional Medical Center, 4513 Akasegawa, Akune 899-1611, Japan (YT, NK); Division of Medical and Environmental Safety, Kagoshima University Medical and Dental Hospital, 8-35-1 Sakuragaoka, Kagoshima 890-8520, Japan (KY); and Physical Therapy Department, School of Health Sciences, Faculty of Medicine, Kagoshima University, 8-35-1 Sakuragaoka, Kagoshima 890-8544, Japan (KY).

## Abstract

The efficacy and safety of chemical prophylaxis to prevent the development of deep venous thrombosis (DVT) or pulmonary embolism (PE) following spine surgery are controversial because of the possibility of epidural hematoma formation. Postoperative venous thromboembolism (VTE) after spine surgery occurs at a frequency similar to that seen after joint operations, so it is important to identify the risk factors for VTE formation following spine surgery. We therefore retrospectively studied data from patients who had undergone spinal surgery and developed postoperative VTE to identify those risk factors.

We conducted a retrospective clinical study with logistic regression analysis of a group of 80 patients who had undergone spine surgery at our institution from June 2012 to August 2013. All patients had been screened by ultrasonography for DVT in the lower extremities. Parameters of the patients with VTE were compared with those without VTE using the Mann–Whitney U-test and Fisher exact probability test. Logistic regression analysis was used to analyze the risk factors associated with VTE. A value of *P* < 0.05 was used to denote statistical significance.

The prevalence of VTE was 25.0% (20/80 patients). One patient had sensed some incongruity in the chest area, but the vital signs of all patients were stable. VTEs had developed in the pulmonary artery in one patient, in the superficial femoral vein in one patient, in the popliteal vein in two patients, and in the soleal vein in 18 patients. The Mann–Whitney U-test and Fisher exact probability test showed that, except for preoperative walking disability, none of the parameters showed a significant difference between patients with and without VTE. Risk factors identified in the multivariate logistic regression analysis were preoperative walking disability and age.

The prevalence of VTE after spine surgery was relatively high. The most important risk factor for developing postoperative VTE was preoperative walking disability. Gait training during the early postoperative period is required to prevent VTE.

## Abstract 2

Supplemental Digital Content is available in the text

## INTRODUCTION

It has been recommended that chemical prophylaxis be used after total knee or total hip arthroplasty or after open reduction/internal fixation of proximal hip fractures to prevent the development of deep venous thrombosis (DVT) or pulmonary embolism (PE). The efficacy and safety of chemical prophylaxis following spine surgery, however, are controversial because of the possibility of epidural hematoma formation. The North American Spine Society Clinical Guideline (Antithrombotic Therapies in Spine Surgery) reported that the utility and safety of chemoprophylaxis have not been thoroughly studied even in high-risk patients undergoing spinal surgery for traumatic or neoplastic conditions. Postoperative venous thromboembolism (VTE) after spine surgery, however, occurs at a frequency similar to that seen after joint operations. Thus, it is important to identify the risk factors for VTE formation after spine surgery. We based our study on this premise.

## MATERIALS AND METHODS

We retrospectively evaluated 80 patients who had undergone spine surgery at our institution from June 2012 to August 2013. These patients (41 women, 39 men) ranged in age from 22 to 85 years (mean 66.2 years). We had performed preoperative lower extremity ultrasonography in patients whose D-dimer concentration was >1.0 μg/mL. When a VTE was found preoperatively at our institution, an anticoagulant was administered until 6 hours prior to surgery. All patients in this study had been screened by ultrasonography for DVT of the lower extremities 7 days after their surgery. One of the patients had sensed some incongruity in the chest, so we evaluated it using contrast-enhanced computed tomography (CT) to look for a PE. Mechanical methods, such as compression stockings and sequential pneumatic compression, had been used for prophylaxis against VTE in all of the patients. We undertook anticoagulation in the patients in whom DVT was found.

For the present study, the parameters of the patients with VTE were compared with those without VTE using the Mann–Whitney U-test and Fisher exact probability test. The Mann–Whitney U-test was used for numerical data (eg, patient's age, operation time, blood loss, D-dimer, days before walking after surgery, white blood cell count, body mass index). Fisher exact probability test was used to identify differences in the expected versus observed frequency of nominal variables (eg, sex, preoperative walking disability, hypertension, diabetes mellitus, smoking, anticoagulant use). Statistical significance was defined as *P* < 0.05.

Logistic regression analysis was applied to identify the risk factors associated with VTE. A value of *P* < 0.05 was used to denote statistical significance. The software used for analyses was IBM SPSS Statistics version 21.0 (IBM, Armonk, NY, USA). The local ethics committee of Izumi Regional Medical Center reviewed and approved this study (20140919-1).

## RESULTS

The records indicated that we had performed lower extremity ultrasonography in patients whose D-dimer concentrations were >1.0 μg/mL preoperatively and had identified two cases of DVT. The postoperative prevalence of VTE was 25% (20/80 patients). We excluded two patients who were found to have a DVT preoperatively. One patient had sensed some incongruity in the chest. CT examination found a PE, although the vital signs were stable. The other 19 patients were clinical asymptomatic.

The median ages for the patients with VTE and those without VTE were 75.0 and 70.5 years, respectively; median operation times were 212.5 and 177.5 minutes; median blood losses were 110.0 and 60.0 g; and median preoperative D-dimer concentrations were 0.7 and 0.5, respectively. Based on the Mann–Whitney U-test results and Fisher exact probability test, none of the measured parameters—except preoperative walking disability—showed significant differences between patients with and without VTE. Initiation of gait training occurred later in the VTE group (Table 1). In all, one patient developed VTE in the pulmonary artery, one in the superficial femoral vein, two in the popliteal vein, and 18 in the soleal vein. Most of the VTEs were located distally (Table 2). Operation time and blood loss did not show significant differences between patients with and without VTE according to the VTE site using the Mann–Whitney U-test (Table 3). DVTs were confirmed preoperatively in two patients. We prescribed anticoagulation for the patients whose DVTs were diagnosed more than 7 days after the operation. There were no cases of paralysis caused by epidural hematomas in our study.

**TABLE 1:**
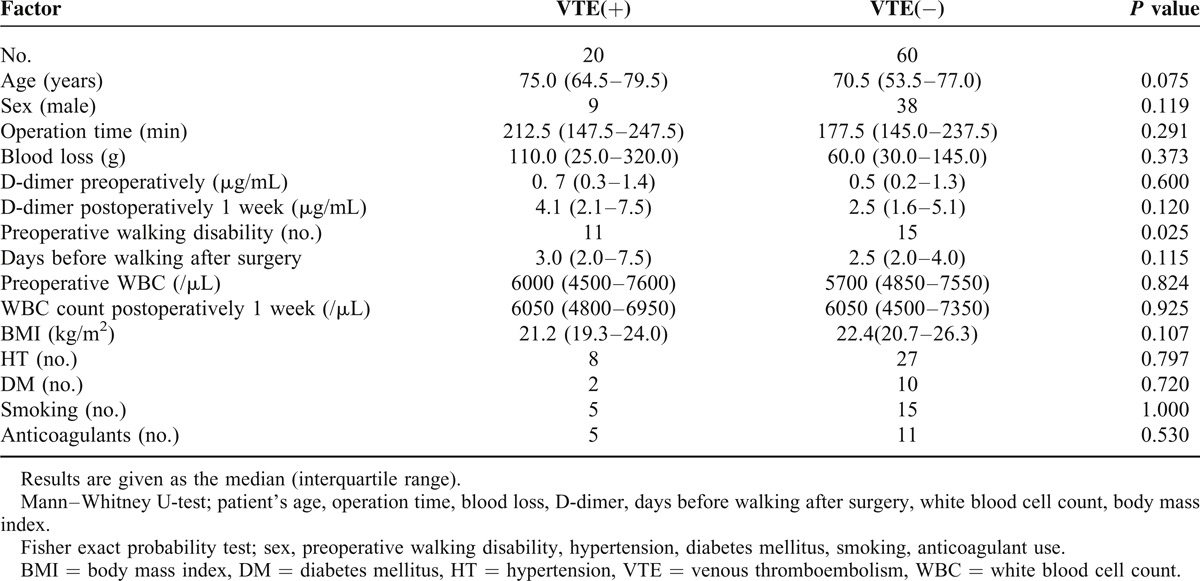
Patient Characteristics

**TABLE 2:**
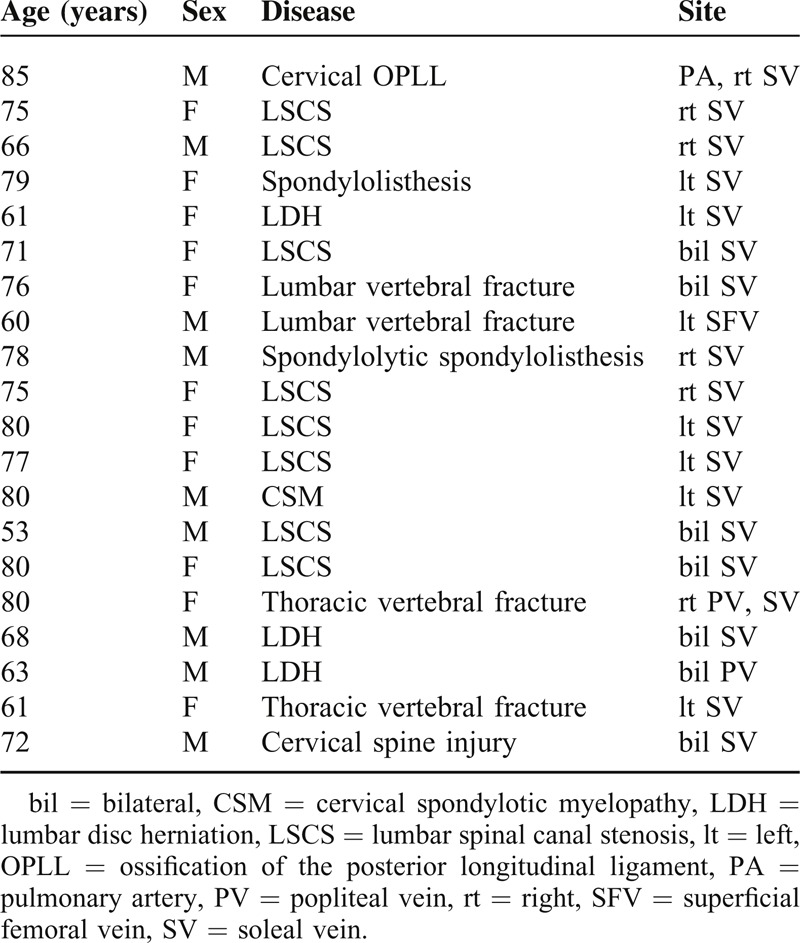
Details of Patients With VTE

**TABLE 3:**
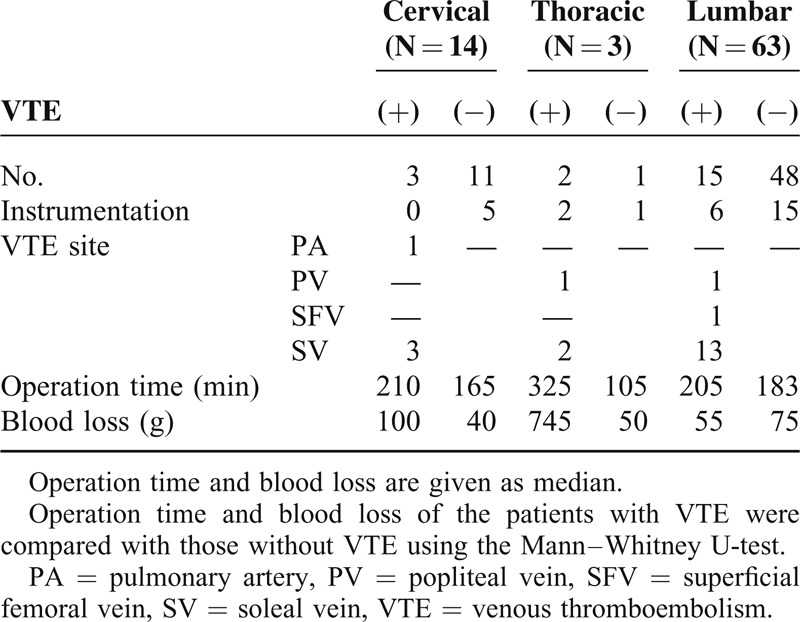
VTE Characteristics

**FIGURE 1:**
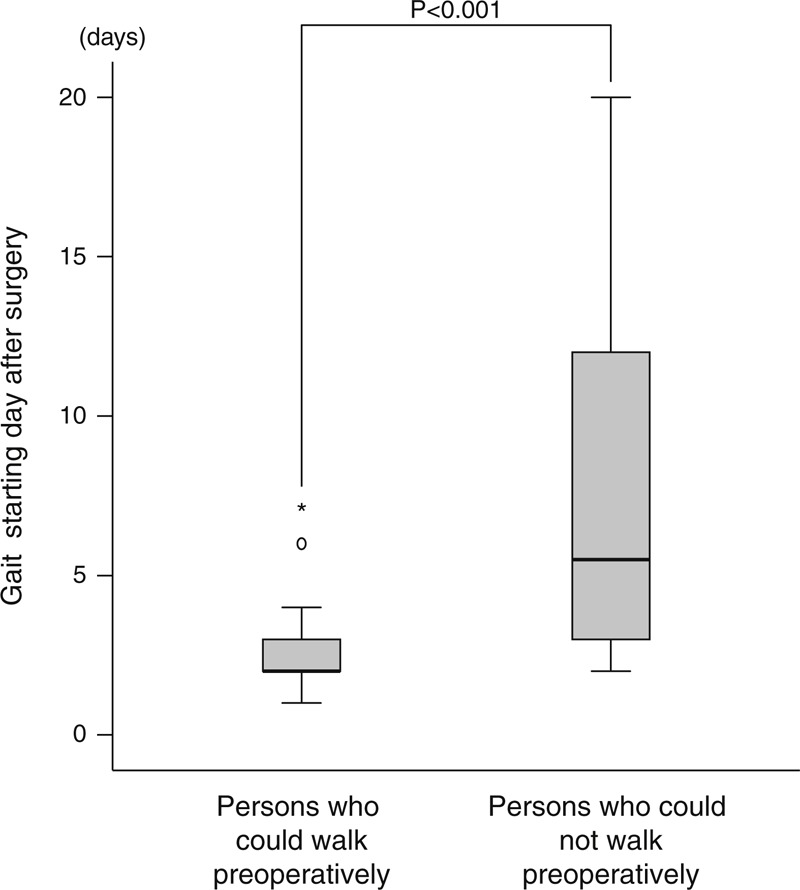
Individuals who had a walking disability preoperatively starting walking significantly later after surgery than those who had no preoperative walking disability. Statistics by Mann-Whitney U-test.

The multivariate logistic regression analysis identified two risk factors: preoperative walking disability (OR 4.829, 95% CI 1.534–15.204) and age (OR 1.058, 95% CI 1.007–1.112) (Table 4). Individuals who had a walking disability preoperatively started walking significantly later after surgery than those who had no preoperative walking disability (Figure 1).

**TABLE 4:**
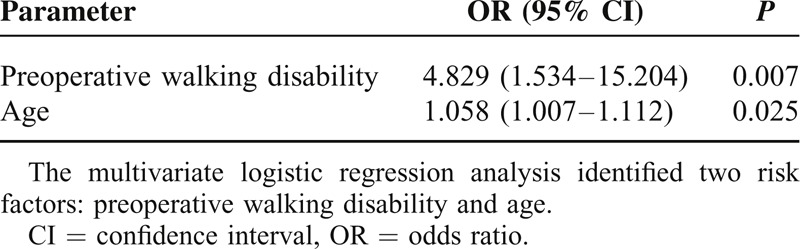
Multivariate Analysis (Logistic Regression)

Supplement Table. http://links.lww.com/MD/A177

## DISCUSSION

There are some published reports regarding the risk factors for DVT development in patients who undergo spine surgery. Takahashi et al^1^ performed a radiological study using contrast-enhanced CT to evaluate 100 patients after spine surgery. They reported asymptomatic PE and DVT in 18% and 19% of patients, respectively. Hohl et al^2^ conducted a multi-institutional study of patients who underwent spine surgery and reported that the prevalence of VTE was 1.5%, symptomatic PE 0.88%, and DVT 0.66%. They also reported that patients >65 years at the time of surgery had a 2.196 times higher prevalence of DVT and PE. These authors found no statistical significance for sex, instrumentation, or revision surgery as risk factors. Schoenfeld et al^3^ identified a body mass index of ≥40 kg/m^2^, age ≥80 years, operation time >261 minutes, and American Society of Anesthesiologists classification of ≥3 as significant independent predictors of DVT.

To prevent VTE development, it has been recommended that chemical prophylaxis be applied after total knee and total hip arthroplasties and after open reduction/internal fixation of proximal hip fractures. Nagase et al^4^ reported that fondaparinux in combination with mechanical prophylaxis prevents postoperative PE after total hip or knee arthroplasty.

Epidural hematoma is a serious complication after spine surgery, resulting in spinal paresis. The prevalence of symptomatic postoperative epidural hematoma is in the range of 0.1%–3.0%.^5–7^ Strom and Frempong-Boadu^8^ reported that low-molecular-weight heparin (LMWH) prophylaxis seems to be associated with a low risk of hemorrhage when started 24–36 hours after spine surgery. Zhi-jian et al^9^ reported that their patients were given a half dose of LMWH 6 hours after spine surgery followed by a full dose of LMWH once a day until discharge. There were no major bleeding events in their patients. Sansone et al^10^ reported that the use of pharmacological prophylaxis significantly reduced the prevalence of DVT compared with mechanical prophylaxis or no prophylaxis. Anticoagulant therapy was performed infrequently during the early postoperative period.^11,12^ Close neurological monitoring is recommended when using chemoprophylaxis because it increases the risk of hematoma.^12^ Schizas et al reported that LMWH was not effective in reducing the prevalence of PE.^13^

Although the prevalence of VTEs after spinal surgery is relatively high, there are many negative opinions about administering anticoagulant therapy during the early postoperative period. Alhalbouni et al reported that there was no statistically significant difference in the risk of PE between isolated femoropopliteal and isolated infrapopliteal DVTs, and hospitalized patients with infrapopliteal DVTs should receive anticoagulation.^14^ Deitcher and Caprini reported that symptomatic calf DVT should be treated with anticoagulation.^15^ On the other hand, Rokito et al reported that anticoagulation is no more effective than mechanical prophylaxis for reducing DVT risk.^16^ Schwarz et al concluded that their data did not show superiority of a short-term regimen of LMWH and compression therapy when compared with compression therapy alone in patients with isolated calf muscle vein thrombosis in a rather low-risk population.^17^

This study has limitations. First, we report on only a small number of patients. Also, we did not perform CT venography or CT pulmonary angiography in all patients because we wanted to prevent side effects and reduce medical costs. Therefore, it is possible that small PEs might not have been identified. In addition, we did not include patients who were not anticoagulated. Our findings therefore do not allow us to recommend chemical prophylaxis.

Although most of the DVTs in this study were located in the soleal vein, our findings showed that they also developed in the pulmonary artery, superficial femoral vein, and popliteal vein following spine surgery. These findings suggest that DVT prevention by chemical prophylaxis might be needed in high-risk patients, especially those with preoperative walking disability, during the perioperative period.

It is important to identify risk factors for VTE that develop after spine surgery. Although it has been reported that early gait training is important for preventing VTE, it takes a long time for muscular strength to improve to the extent required for walking. Even when neurological symptoms recede in DVT patients, those who could not walk well preoperatively have problems walking postoperatively.

Recently, neurological rehabilitation using a robot suit for patients with paralysis has been reported. Aach et al^18^ reported that training with the Hybrid Assistive limb (HAL®) exoskeleton results in improved ground walking. This report leads to the assumption that there is a beneficial effect on ambulatory mobility in patients with a chronic spinal cord injury. We also have reported that early mobilization using HAL may be advocated to prevent postoperative complications, such as contractures and DVT.^19^ HAL usage was recognized as an acceptable treatment under workmen's accident compensation insurance in Germany in 2013.

Many elderly people undergo spine operations. It is extremely important that patients at high risk for developing VTE—especially those with preoperative walking disability or postoperative walking disability caused by muscle weakness—should begin gait training as soon as possible after surgery.
